# Early Social Isolation Stress and Perinatal NMDA Receptor Antagonist Treatment Induce Changes in the Structure and Neurochemistry of Inhibitory Neurons of the Adult Amygdala and Prefrontal Cortex

**DOI:** 10.1523/ENEURO.0034-17.2017

**Published:** 2017-05-01

**Authors:** Esther Castillo-Gómez, Marta Pérez-Rando, María Bellés, Javier Gilabert-Juan, José Vicente Llorens, Héctor Carceller, Clara Bueno-Fernández, Clara García-Mompó, Beatriz Ripoll-Martínez, Yasmina Curto, Noelia Sebastiá-Ortega, María Dolores Moltó, Julio Sanjuan, Juan Nacher

**Affiliations:** 1 Neurobiology Unit, Cell Biology Department, Interdisciplinary Research Structure for Biotechnology and Biomedicine (BIOTECMED), Universitat De València, Burjassot 46100, Spain; 2 Department of Genetics, Universitat De València, Burjassot 46100, Spain; 3 CIBERSAM: Spanish National Network for Research in Mental Health, 28029 Madrid; 4 Fundación Investigación Hospital Clínico De Valencia, INCLIVA, Valencia 46010, Spain

**Keywords:** interneuron, neuronal plasticity, PSA-NCAM, schizophrenia, social isolation, stress

## Abstract

The exposure to aversive experiences during early life influences brain development and leads to altered behavior. Moreover, the combination of these experiences with subtle alterations in neurodevelopment may contribute to the emergence of psychiatric disorders, such as schizophrenia. Recent hypotheses suggest that imbalances between excitatory and inhibitory (E/I) neurotransmission, especially in the prefrontal cortex and the amygdala, may underlie their etiopathology. In order to understand better the neurobiological bases of these alterations, we studied the impact of altered neurodevelopment and chronic early-life stress on these two brain regions. Transgenic mice displaying fluorescent excitatory and inhibitory neurons, received a single injection of MK801 (NMDAR antagonist) or vehicle solution at postnatal day 7 and/or were socially isolated from the age of weaning until adulthood (3 months old). We found that anxiety-related behavior, brain volume, neuronal structure, and the expression of molecules related to plasticity and E/I neurotransmission in adult mice were importantly affected by early-life stress. Interestingly, many of these effects were potentiated when the stress paradigm was applied to mice perinatally injected with MK801 ("double-hit" model). These results clearly show the impact of early-life stress on the adult brain, especially on the structure and plasticity of inhibitory networks, and highlight the double-hit model as a valuable tool to study the contribution of early-life stress in the emergence of neurodevelopmental psychiatric disorders, such as schizophrenia.

## Significance Statement

The double-hit model constitutes a valuable tool for future experiments exploring the effects of aversive experiences during early life and the biological basis of mental disorders, such as schizophrenia. It also supports the emerging hypothesis of altered E/I balance in key brain regions as one of the underlying causes of the disease. Our study also supports the idea that such imbalances may arise from problems in initial neural circuit formation or its maintenance, because we found alterations in the structure of inhibitory circuits and also in the expression of molecules related to their plasticity and maturation.

## Introduction

Aversive experiences, such as chronic stress, remodel the structure and connectivity of excitatory and inhibitory excitatory and inhibitory (E/I) neurons. These effects of stress are particularly relevant during early life and may constitute a predisposing factor for the development of psychiatric disorders, such as schizophrenia. In fact, patients show important alterations in different brain regions, including the prefrontal cortex (PFC) and the amygdala (Goghari et al. 2010). Interestingly, the structure of E/I neurons in these two regions is dramatically affected by stress (Gilabert-Juan et al. 2011, 2013b; Duman and Duman, 2015).

Different animal models have been generated to understand the impact of early-life stress on the structure of the adult brain and its influence on schizophrenia and other psychiatric disorders. The post-weaning social isolation paradigm is one of these models and reproduces some of the behavioral, structural, and neurochemical alterations found in schizophrenic patients (Geyer et al. 1993; Ferdman et al. 2007; Gilabert-Juan et al. 2012; Wang et al. 2012; Glausier and Lewis, 2013). Given the importance of altered neurodevelopment on the etiology of schizophrenia and other mental disorders, this paradigm has been lately combined with experimental interventions during early postnatal life, such as the administration of NMDA receptor (NMDAR) antagonists (i.e., MK801), which alters the latest stages of neocortical development (Hickey et al. 2012; Lim et al. 2012; Gilabert-Juan et al. 2013a). Numerous studies in humans and animal models of schizophrenia, have documented the presence of structural alterations in the basolateral amygdala (BLA) and PFC, including volume loss in both regions (Jaaro-Peled et al. 2010; Levitt et al. 2010; Gilabert-Juan et al. 2013a). These volumetric alterations may probably reflect structural changes in PFC and BLA neurons, including alterations in spine density and dendritic arborization.

The polysialylated form of the neural cell adhesion molecule (PSA-NCAM) plays a key role in structural remodeling and the connectivity of neurons in the adult brain, especially of interneurons (for review, see Bonfanti, 2006; Rutishauser, 2008; Nacher et al. 2013), which is particularly evident after chronic stress (Sandi, 2004; Nacher et al. 2013). Perineuronal nets (PNNs) also play a fundamental role in the plasticity and maturation of interneurons, particularly on those expressing parvalbumin (PV; Kinden Lensjø et al. 2017). In fact, this plasticity of inhibitory networks is crucial for brain physiology and development, which largely depend on the precise balance between E/I neurotransmission (E/I balance). The E/I balance is compromised by stress, both in adulthood and in early life (Saaltink and Vreugdenhil, 2014; Tzanoulinou et al. 2014; van der Kooij et al. 2014) and its disbalance may be one of the underlying causes of different neurodevelopmental psychiatric disorders, including schizophrenia (Curley and Lewis, 2012; Lewis et al. 2012; Inan et al. 2013; Lin et al. 2013; Sun et al. 2013; Morishita et al. 2015). Importantly, E/I balance can also be affected by changes in the expression of other molecules that influence the physiology and development of inhibitory circuits. This is the case of glutamic acid decarboxylase (GAD; Akbarian, 1995; Akbarian and Huang, 2006; Straub et al. 2007; Mitchell et al. 2015); brain-derived neurotrophic factor (BDNF), which promotes the maturation of inhibitory synapses (Weickert et al. 2003, 2005; Tao et al. 2014); the cannabinoid receptor 1 (CB1-R), which affects their development (Volk and Lewis, 2010; den Boon et al. 2015); and neuregulin 1 (Nrg1) and its receptor ErbB4, which play prominent roles in the synaptogenesis and plasticity of inhibitory networks (Rico and Marín, 2011).

The main objective of this work is to study the impact of early-life stress on E/I circuits in the adult amygdala and PFC, especially on neuronal structure. To combine this aversive experience with a neurodevelopmental alteration, which mimic those found in schizophrenia, we have developed a "double-hit" model in transgenic mice by combining a perinatal MK801 injection and post-weaning social isolation. We have used two transgenic strains expressing fluorescent proteins in E/I neurons and studied in detail the structural features of these neurons, the expression of molecules related to plasticity and E/I neurotransmission and the behavior of the animals.

## Materials and Methods

### Animals and Experimental Treatment

**Figure 1.**
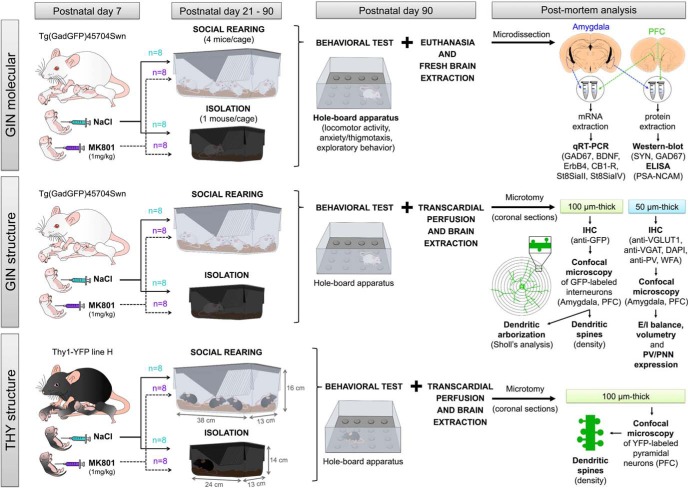
Experimental procedure. Seven days after birth (P7), male pups from two different transgenic strains: the GIN mice (Oliva et al. 2000), which express EGFP in interneurons, and the THY1 mice (Feng et al. 2000), which express the YFP in pyramidal neurons) were intraperitoneally injected with MK801 or NaCl (vehicle solution). After weaning (P21), mice were randomly selected and housed alone (isolation) or in groups of four mice (social rearing) for 10 weeks. At P90, all mice were tested in the hole-board apparatus. Brains from GIN molecular mice were destined to protein and gene expression studies, whereas brains from GIN structure mice were used for the structural and neurochemical analysis of interneurons. The THY structure set of mice was used to study structural alterations of pyramidal neurons. For further details, see Material and Methods.

Two different transgenic mice strains, purchased from The Jackson Laboratory were used in our experiments: the GIN mice [EGFP-expressing inhibitory neurons, Tg(GadGFP)45704Swn], which express the enhanced green fluorescent protein (EGFP) in a subpopulation of interneurons of interest (Oliva et al. 2000); and the THY1 mice (Thy1-YFP line H), which express the yellow fluorescent protein (YFP) in a subset of pyramidal neurons (Feng et al. 2000). The experimental procedure was performed twice for the GIN mice and once for the THY1 mice (Fig. 1). One set of the GIN mice (*n* = 32) was used for the structural and neurochemical analysis of interneurons (“GIN structure”), and the other set (*n* = 32) was used for molecular analyses (“GIN molecular”). All THY mice (*n* = 32) were used for the structural analysis of pyramidal neurons (“THY structure”). The following procedure refers to any of the three sets. Breeding cages containing one male and two female mice (3 months old) were maintained in our animal facility under standard conditions of temperature and light (12 h light/dark cycle) and ad libitum access to food and water. Once the females were pregnant, they were housed individually to avoid any disturbances among mice. Seven days after birth (P7), male pups received randomly a single intraperitoneal injection of MK801 (1 mg/kg solved in NaCl 0.9%, Abcam) or the vehicle solution (NaCl 0.9%). This dose and age of administration produced alterations in PFC-dependent behavior and changes in the structure and inhibitory networks of this region (Lyall et al. 2009; Gilabert-Juan et al. 2013a). MK801, also known as dizocilpine, is a noncompetitive antagonist of NMDA receptors. After the injection, pups were returned to their cages and remained with their mother until the age of weaning (P21). At this age, eight mice from each of the former groups (NaCl or MK801) were randomly selected and housed alone (social isolation) in small polycarbonate cages (24 × 14 × 13 cm; Zoonlab-Bioscapey) or in groups of three to four mice (social housing) in standard-size cages (38 × 16 × 13 cm; Zoonlab-Bioscape) for 10 weeks (P90). Thus, at this point, the four final experimental groups (*n* = 8 mice/group) were determined: NaCl-Social (injected with vehicle at P7, and socially housed after weaning), NaCl-Isolation (injected with vehicle but isolated after weaning), MK801-Social (injected with MK-801 at P7 and reared in group), and MK801-Isolation or double-hit model (injected with MK801 at P7 and isolated after weaning). All mice were housed in the same room, sharing the same controlled-environment. Isolated mice were able to hear and smell other mice but physical or visual contact with them was not allowed (Fig. 1).

All animal experimentation was conducted in accordance with the Directive 2010/63/EU of the European Parliament and of the Council of 22 September 2010 on the protection of animals used for scientific purposes and was approved by the Committee on Bioethics of the Universitat de València. Every effort was made to minimize the number of animals used and their suffering.

### Behavioral Analyses

**Figure 2.**
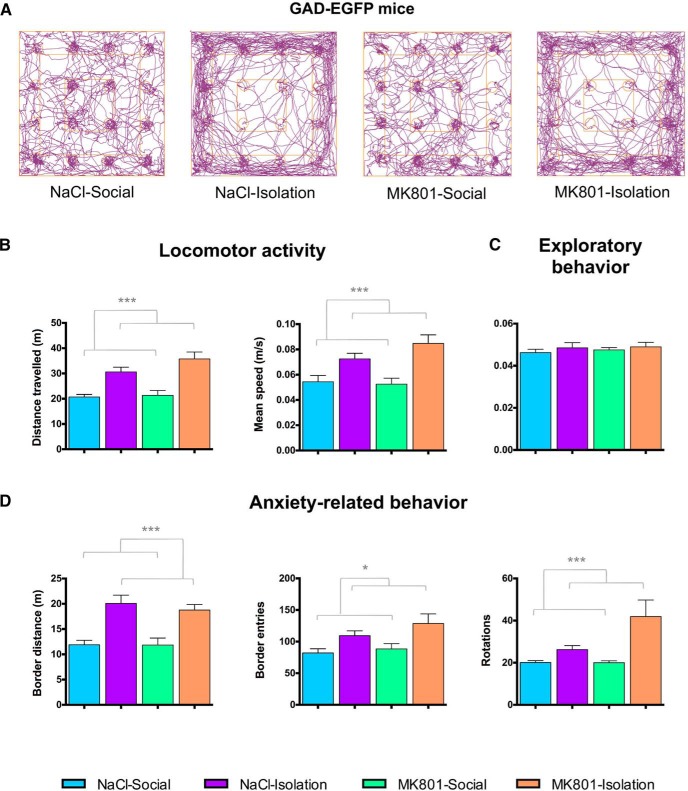
Behavioral analysis of GAD-EGFP expressing mice. ***A***, Representative track-plot reports recorded during the hole-board test session (ANY-maze). Observe the increased distance traveled (purple line) in the two groups subjected to post-weaning isolation, especially in the border of the apparatus. Social isolation rearing increased locomotor activity (***B***) and anxiety-related behavior (***D***) but did not change exploratory behavior (number of head dips into novel holes/total number of head dips; ***C***). Gray lines in graphs (***B*–*D***) represent statistically significant effects of rearing in a two-way ANOVA. *p < 0.05, **p < 0.01, ***p < 0.001.

**Figure 3.**
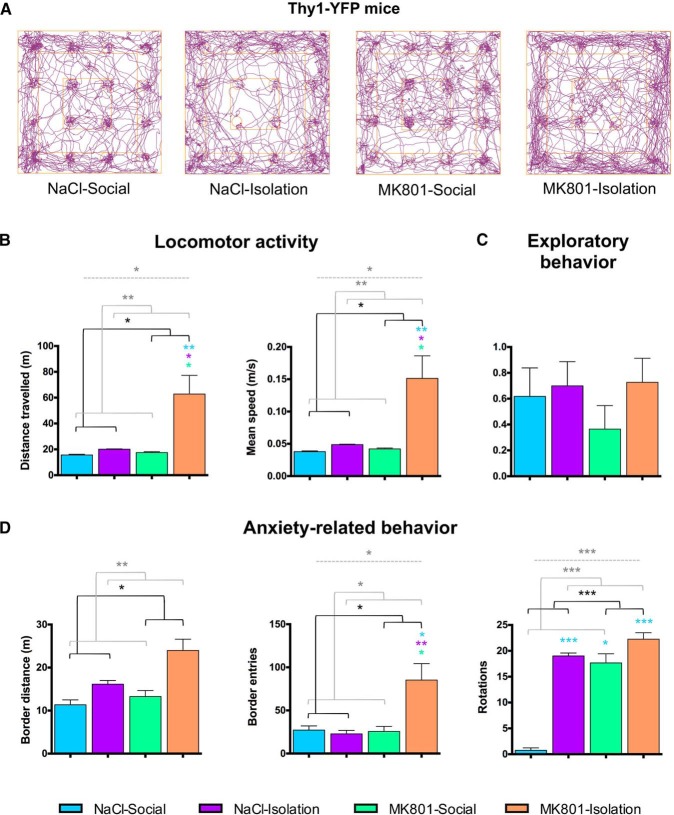
Behavioral analysis of Thy1-YFP expressing mice. ***A***, Representative track-plot reports showing the increased distance traveled (purple line) of double-hit mice (MK801-Isolation group) in the hole-board test compared with the other groups. Double-hit mice showed increased locomotor activity (***B***) and anxiety-related behavior (***D***) but no changes in exploratory behavior (number of head dips into novel holes/total number of head dips; ***C***). ***B***–***D***, Horizontal lines in graphs represent statistically significant effects of MK801 treatment (black), rearing (gray), or interaction (gray, dashed) in a two-way ANOVA. *p < 0.05, **p < 0.01, ***p < 0.001. Colored symbols in bars represent trends and statistically significant differences among groups after *post hoc* analysis: # 0.1 > *p* > 0.05, *p < 0.05, **p < 0.01, ***p < 0.001.

Before their sacrifice (P90), all mice were tested in the hole-board apparatus (ANY-maze video tracking system v4.98; Stoelting Europe). The hole-board test measures directed exploration but can also be used as an initial basic screen for working memory (Karl et al. 2008), locomotor activity, and anxiety-related behavior (Castilla-Ortega et al. 2010; Torres-García et al. 2012; Fig. 1). The open-field chamber (40 × 40 cm) was fitted with a hole-board floor insert for mice (16 holes, diameter = 2.8 cm, non-baited). Testing of male mice took place between 1 and 2 h after the onset of the dark phase (illumination at floor level <2 lx). Each mouse was placed in the center of the arena and was left to explore the environment for 7 min (test session). The video tracking system and the infrared photobeams provided automated measures of the following: (1) total distance traveled and mean speed (to study locomotor activity); (2) head dips, for the study of exploratory behavior (number of head dips into novel holes/total number of head dips; Karl et al. 2008); and (3) body rotations (360°) and number of entries and time spent in the periphery of the arena, for the measure of anxiety and thigmotaxis (a valid index of anxiety in mice; Simon et al. 1994). The periphery zone of the area was defined as the area located between 0 and 6 cm away the walls of the apparatus (Figs. 1,2A,3A).

### Perfusion, Microtomy, and Immunohistochemistry

At P90, GIN structure and THY structure mice were deeply anesthetized with sodium pentobarbital and transcardially perfused with 4% paraformaldehyde solution for 20 min. Thirty minutes after perfusion, brains were extracted from the skull and their hemispheres were separated. One hemisphere was cryoprotected [30% sucrose in phosphate buffer (PB) 0.1m, 48 h] and afterward cut in 50-µm-thick coronal sections using a freezing-sliding microtome (LEICA SM2000R, Leica) for immunohistochemical analysis. The other hemisphere, destined to the study of the neuronal structure, was cut in 100-μm-thick coronal sections with a vibratome (Leica VT 1000E, Leica; Fig. 1).

After cutting, tissue was counterstained with DAPI (50-μm-thick sections for volumetric studies; see below) or processed free-floating for fluorescence immunohistochemistry as follows (Fig. 1). Briefly, sections were washed in PBS and then incubated for 1 h in 10% normal donkey serum (NDS; Abcys) in PBS with 0.2% Triton X-100 (PBST; Sigma-Aldrich). Afterward, they were incubated for 48 h at 4°C with the appropriate primary antibody or antibody cocktail diluted in PBST and 5% NDS: (a) polyclonal chicken IgY anti-GFP (1:1000, Abcam) in 100-μm-thick sections from GIN mice to enhance EGFP signal for the structural study of interneurons; (b) polyclonal guinea pig anti-vesicular glutamate transporter 1 (VGLUT1; 1:2000, Merck-Millipore) and monoclonal IgG mouse anti-vesicular GABA transporter (VGAT; 1:1000, Synaptic Systems) in 50-μm-thick sections to study excitatory and inhibitory neurotransmission in the neuropil; (c) polyclonal rabbit IgG anti-PV (1:2000, Swant) and biotin-conjugated *Wisteria floribunda* agglutinin (1:200, Sigma-Aldrich) in 50-μm-thick sections to study the coexpression of PV and PNNs in the amygdala and PFC. After washing, sections were incubated for 2 h at room temperature with matching secondary antibodies (1:400, Jackson ImmunoResearch) diluted in PBST and 5% NDS: (a) donkey anti-chicken DyLight-488-conjugated, (b) goat anti-guinea Pig DyLight-649-conjugated and goat anti-mouse IgG AlexaFluor-555-conjugated, and (c) donkey anti-rabbit IgG AlexaFluor-555-conjugated and Avidin AlexaFluor-647-conjugated. Finally, sections were washed in PB 0.1 m, mounted on slides, and coverslipped using fluorescence mounting medium (Dako Diagnósticos).

### Volumetry

A volumetric analysis of the different nuclei of the amygdala (central, lateral, medial, basolateral, and basomedial) and regions of the PFC (infralimbic, prelimbic, dorsal cingulate, and ventral cingulate cortices) were performed by processing confocal images using the Volumest plugin of FIJI/ImageJ Software (NIH; Schindelin et al. 2012). To be able to differentiate the different regions of interest in the subsets of slices, 50-μm-thick sections from the GIN structure set of mice (*n* = 32) were counterstained with DAPI (1:10,000 in H_2_O, Sigma-Aldrich). Images of the regions-of-interest were acquired using a confocal microscope (Olympus FV-10). Volumes were estimated using Cavalieri's principle (Gundersen and Jensen, 1987).

### Analysis of Dendritic Arborization and Dendritic Spine Density

All the structural parameters of the GAD-EGFP interneurons and THY1-YFP pyramidal neurons were studied using a laser scanning confocal microscope (Leica TCS SPE). In the case of pyramidal neurons, we focused our studies on the PFC, in particular, on the cingulated cortices because, due to the straight projection of the principal dendrite of pyramidal neurons to layer 1, it is only visible coronally in these two subregions of the PFC. To be consistent, interneurons were studied in the same subregions of the PFC. In addition, we studied the arborization and dendritic spine density of interneurons in the basolateral nucleus of the amygdala, which is known to project directly to the PFC and has an abundant population of fluorescent interneurons in GIN mice. Unfortunately, due to the overwhelming expression of the YFP in the amygdala, we could not to perform these structural analyses for pyramidal neurons.

For the study of the dendritic arborization, six GAD-GFP expressing interneurons per animal and region were randomly selected (Fig. 1). Z-series of optical sections (0.8 μm step size; 40× objective) covering the dendritic tree of selected interneurons were obtained using the sequential scanning mode. To be suitable for analysis, these interneurons had to fulfill the following features: (1) the cell must not show any truncated dendrites, (2) the dendritic arbor of the cell must show at least a process with a length >150 μm, and (3) the soma must be located at least 30 μm deep from the surface of the tissue. The stacks obtained were then processed using FIJI (ImageJ, NIH) software to render 3D reconstructions. Neurons were traced using the “Simple neurite tracer” plugin, which also allowed us to analyze their Sholl profile in 3D (Longair et al. 2011; Fig. 1). The Sholl analysis consists on the measure of the number of intersections of the dendrites with spheres of increasing radius centered in the soma. The separation among the spheres of the analysis was set at 20 μm. For each animal, mean ± SEM was calculated and statistics were performed using the number of animals and the sample number (*n*; see below).

For the analysis of dendritic spines, six GAD-GFP expressing interneurons and six THY1-YFP expressing pyramidal neurons per animal and region were randomly selected (Fig. 1). A 63× oil immersion objective and a 3.5× additional digital zoom were used to observe the first 150 µm of the dendrite in the case of interneurons and the first 200 µm of the dendrite in the case of pyramidal neurons in segments of 50 µm (Z-step size of 0.38 µm). Dendrites within EGFP- and YFP-positive interneurons were randomly selected, but they had to meet the following criteria to be included in the study: (1) their length should be at least 150 µm (for interneurons) or 200 µm (for pyramidal neurons), and (2) no other dendrites should be found crossing their trajectory. For interneurons, data were expressed as the total number of spines in the proximal (0–50 µm), medial (50–100 µm), and distal (100–150 µm) segments of the dendrite, depending on its distance from the soma. For pyramidal neurons, four segments were established: proximal (0–50 µm), medial (50–100 µm), medial-distal (100–150 µm), and distal (150–200 µm). The total number of spines in every dendrite (sum of the spines in the entire segment) was also analyzed. For each animal, mean ± SEM was calculated and statistics were performed using the number of animals and the sample number (*n*; see below).

### Analysis of VGLUT1 and VGAT Puncta Density in the Neuropil and Calculation of the E/I Ratio

The images used for the analysis of neuropil puncta expressing inhibitory (VGAT) or excitatory (VGLUT1) markers were obtained with a confocal microscope (Olympus FV-10). We analyzed layer 5 of the different regions of the PFC (infralimbic, prelimbic, dorsal cingulate, and ventral cingulate cortices). In the amygdala, the five nuclei were analyzed (central, lateral, medial, basolateral, and basomedial). Confocal *z*-stacks covering the whole depth of the sections were taken with 1 μm step size and only subsets of confocal planes with the optimal penetration level for each antibody were selected. On these planes, small regions of the neuropil (505 μm^2^) were selected for analysis to avoid blood vessels and cell somata. Images were processed using FIJI/ImageJ software as described (Guirado et al. 2012): the background was subtracted with rolling value of 50, converted to 8-bit deep images and binarized using a determined threshold value. This value depended on the marker and the area analyzed and was kept the same for all images with the same marker and area. Then, the images were processed with a blur filter to reduce noise and to separate closely apposed puncta. Finally, the number of the resulting dots per region was counted. The E/I ratio was calculated as the density of VGLUT1 expressing puncta divided by the density of inhibitory VGAT expressing puncta. For each animal, mean ± SEM was calculated and statistics were performed using the number of animals and the sample number (*n*; see below).

### Estimation of the Total Number of PV-Expressing Neurons, PNN, and PV-PNN Colocalization

The total number of PV-expressing (PV+) neurons, PNNs, and PV+ neurons surrounded by PNNs from the different nuclei/regions of the amygdala and PFC were estimated using a modified version of the fractionator method (West, 1993; Nacher et al. 2002). That is, within each 50-μm-thick section of one from the six systematic-random series of sections, all labeled cells covering the 100% of the sample area were counted. The images used for the analysis were obtained with a confocal microscope (Olympus FV-10) and processed afterward using FIJI/ImageJ software. For each animal, mean ± SEM was calculated and statistics were performed using the number of animals as the sample number (*n*; see below).

### Sample Preparation for Molecular Studies

A total of 32 mice encompassing the four experimental groups (GIN molecular set of mice) were used for protein and gene expression analyses. Mice were sacrificed by decapitation at P90 under deep anesthesia with sodium pentobarbital. Their brains were quickly removed from the skull and the amygdala and PFC from both hemispheres were microdissected and immediately frozen in liquid nitrogen. The samples from one of the hemispheres of every animal were processed for protein extraction and the samples from the other hemisphere for mRNA extraction (Fig. 1).

For protein extraction, tissue was homogenized in 50 μl of lysis buffer using a TissueLyser (Qiagen) and Tungsten carbide beads for 5 min at 50 Hz/s and 4°C. Lysis buffer was made solving one pill of protease inhibitor cocktail (Ref. 04693124001, Roche Applied Science) in 1 ml of PBS-1% Triton X-100. Samples were afterward centrifuged at 16000 × *g* and 4°C for 4 min and supernatant was assessed for the total amount of protein using Bradford reagent at 595 nm with bovine serum albumin as the standard (Sigma-Aldrich).

Total mRNA was extracted from the tissue using TriPure reagent (Roche Applied Science) and following the manufacturer’s instructions. The concentration and purity of total RNA was determined with an Eppendorf BioPhotometer Plus (Eppendorf AG). cDNA synthesis was performed using the Expand Reverse Transcriptase (Roche Applied Science) and oligo-dT primers.

### Protein Expression

#### Quantitative immunoblotting of synaptophysin and GAD67

Twenty micrograms of total protein from each sample was separated on 10% SDS–PAGE and transferred to Hybond enhanced chemiluminescence (ECL) nitrocellulose membranes (GE Healthcare). After saturation of nonspecific sites with blocking buffer (5% nonfat dry milk in PBS-0.025%Tween 20, overnight, 4°C), membranes were probed for 2 h at room temperature with primary antibodies against synaptophysin (1:5000, Sigma-Aldrich), GAD67 (1:1000, Merck-Millipore) or the control protein α-tubulin (1:5000, Sigma-Aldrich), all of them diluted in blocking buffer. After washing with PBS-0.025%Tween 20, membranes were incubated for 2 h with the appropriate secondary horseradish peroxidase-linked antibodies (1:2500, Sigma-Aldrich), and finally developed using ECL detection reagents (Thermo Scientific). Bands were detected using ImageQuant LAS4000 system (GE Health care) and densitometry analysis on every band was calculated using FIJI/ImageJ software (NIH). Synaptophysin and GAD67 densitometry values were normalized to within-lane α-tubulin. Every sample was immunoblotted in duplicate and mean ± SEM was then calculated. Statistics were performed using the number of animals as the sample number (*n*).

#### ELISA for PSA-NCAM assessment

PSA-NCAM protein levels were quantified in the same samples that were used for synaptophysin and GAD67 quantification, by performing commercially available ELISA kits (Eurobio/AbCys). A total volume of 100 μl of each sample was loaded at a concentration of 4 μg/ml per well in duplicates. PSA-NCAM levels (ng PSA/μg of total protein) were calculated according to the manufacturer’s protocol. Statistics were performed using the number of animals and the sample number (*n*).

### Gene Expression

qRT-PCR analyses were performed in triplicate. Specific primers for all genes (Table 1) at a concentration of 240 nm, and 4 µl of cDNA (50 ng) were used. Primers were designed between exons to avoid genomic DNA contamination when possible. *Ywhaz* was used as a reference gene based on the study of Bonefeld et al. (2008). Primers were tested for nonspecific amplification and for the correct amplicon size by electrophoresis in 1.5% agarose gel. qPCR was conducted with the ABI PRISM 7700 Sequence Detector (Applied Biosystems) using SYBR Green PCR master mix (Applied Biosystems), following a 95°C denaturation for 10 min. The reactions were cycled 40 times with a 95°C denaturation for 15 s, and a 60°C annealing step for 1 min. After this, a melt curve was performed to assess the specificity of primers.

**Table 1.** Sequences of gene specific primers and associated amplicon lengths for qRT-PCR

Target gene	Primers	Sequence (5′ → 3′)	Amplicon size*
*GAD67*	Forward Reverse	CTGGAGCTGGCTGAATACCT TCGGAGGCTTTGTGGTATGT	120
*BDNF*	Forward Reverse	GGTTCGAGAGGTCTGACGAC CAAAGGCACTTGACTGCTGA	159
*ErbB4*	Forward Reverse	CAGTCGCCCAGGGTGCAACG GCGAACACTGTGGGGTCGGC	133
*CB1-R*	Forward Reverse	TGTCCCTCACCCTGGGCACC TCCCAGGAGATCGGCCACCG	134
*ST8SiaII*	Forward Reverse	GGCAACTCAGGAGTCTTGCT GTCAGTCTTGAGGCCCACAT	123
*ST8SiaIV*	Forward Reverse	CCTTCATGGTCAAAGGAGGA CCTTCATGGTCAAAGGAGGA	125
*Ywhaz*	Forward Reverse	TTGAGCAGAAGACGGAAGGT GAAGCATTGGGGATCAAGAA	136

* Amplicon length in base pairs.

Relative quantification was performed using the comparative threshold (*C*_t_) method according to the 2^−ΔΔ^*^C^* ^t^ method (Pfaffl, 2001). Changes in gene expression were reported as fold-change relative to controls and the statistics were performed as described below.

### Statistics

Group differences in all our studies were assessed using two-way ANOVA with the number of animals as the sample number (*n*). When interaction between treatment (MK801 vs vehicle) and rearing (social vs isolation) was statistically significant (*p* < 0.05), multiple pairwise comparisons between groups (Tukey HSD or Games–Howell *post hoc* analyses) were performed. Analysis were performed using the statistical package SPSS v22.0 (IBM) and graphs were created using GraphPad Prism 6. Data in the figures are expressed as mean ± SEM and *p* < 0.05 are indicated.

## Results

### Increased Locomotion and Anxiety-Related Behavior

Early-life stress induced hyperlocomotion and increased anxiety-related behavior in GIN mice, whereas exploratory behavior was not affected (Fig. 2; Table 2). Specifically, double-hit animals (MK801-Isolation) and isolated animals showed increased distance traveled, speed, number of body rotations, and thigmotaxis (increased distance traveled in the periphery zone of the apparatus and increased number of entries to this zone; Fig. 2*B*–*D*). Similar results were found when analyzing the behavior of THY1 mice, but in this case, specific significant increases were found in most of the parameters measured for locomotor activity (Fig. 3*A*,*B*) and anxiety-related behavior (Fig. 3*D*) when comparing double-hit to control mice. Exploratory behavior was also not affected in this strain (Fig. 3*C*). Detailed information on statistical tests can be found in Table 2.

**Table 2.** Summary of results (part I)

Parameter	Main effects (two-way ANOVA)			Group differences (from NaCl-Social)		
	Treatment	Rearing	Interaction	NaCl-Isolation	MK801-Social	MK801-Isolation
**Behavior** (Figs. 2, 3)						
Locomotor activity						
Total distance travelled						
*Gad-EGFP mice*	—	✓^(1)^	—	n/a	n/a	n/a
*Thy1-YFP mice*	✓^(2)^	✓^(3)^	✓^(4)^	—	—	↑**
Mean speed						
*Gad-EGFP mice*	—	✓^(5)^	—	n/a	n/a	n/a
*Thy1-YFP mice*	✓^(6)^	✓^(7)^	✓^(8)^	—	—	↑**
Anxiety-related behavior						
Border distance						
*Gad-EGFP mice*	—	✓^(9)^	—	n/a	n/a	n/a
*Thy1-YFP mice*	✓^(10)^	✓^(11)^	—	n/a	n/a	n/a
Border entries						
*Gad-EGFP mice*	—	✓^(12)^	—	n/a	n/a	n/a
*Thy1-YFP mice*	✓^(13)^	✓^(14)^	✓^(15)^	—	—	↑*
Body rotations						
*Gad-EGFP mice*	—	✓^(16)^	—	n/a	n/a	n/a
*Thy1-YFP mice*	✓^(17)^	✓^(18)^	✓^(19)^	↑***	↑*	↑***
Exploratory behavior						
*Gad-EGFP mice*	—	—	—	n/a	n/a	n/a
*Thy1-YFP mice*	—	—	—	n/a	n/a	n/a
**Brain volume** (Fig. 4)						
Amygdala						
Total volume	—	—	#^(20)^	n/a	n/a	n/a
Central	—	—	✓^(21)^	↑*	—	—
Lateral	—	—	—	n/a	n/a	n/a
Basolateral	—	✓^(22)^	—	n/a	n/a	n/a
Basomedial	✓^(23)^	—	—	n/a	n/a	n/a
Medial	—	—	—	n/a	n/a	n/a
PFC						
Total volume	—	#^(24)^	—	n/a	n/a	n/a
IL	—	#^(25)^	—	n/a	n/a	n/a
PrL	—	—	—	n/a	n/a	n/a
Cg1	✓^(26)^	—	✓^(27)^	—	—	↓#
Cg2	—	—	—	n/a	n/a	n/a

Symbols: (✓) statistically significant effect (two-way ANOVA); (—) no statistically significant effect (two-way ANOVA) or difference from NaCl-Social group (*post hoc*); (↑) increase; (n/a) not applicable; (↓) decrease; *p < 0.05; **p < 0.01; ***p < 0.001; #0.10 ≥ *p* ≥ 0.05.

*F* and *p* values: (1) *F*_(1,58)_ = 31.11, *p* < 10^−6^; (2) *F*_(1,11)_ = 8.05, *p* = 0.016; (3) *F*_(1,11)_ = 9.99, *p* = 0.009; (4) *F*_(1,11)_ = 6.74, *p* = 0.025; (5) *F*_(1,59)_ = 19.38, *p* = 4.6 × 10^−5^; (6) *F*_(1,11)_ = 7.80, *p* = 0.018; (7) *F*_(1,11)_ = 9.90, p = 0.009; (8) *F*_(1,11)_ = 6.61, *p* = 0.026; (9) *F*_(1,59)_ = 36.48, *p* < 10^−7^; (10) *F*_(1,11)_ = 8.22, *p* = 0.015; (11) *F*_(1,11)_ = 20.68, *p* = 0.001; (12) *F*_(1,60)_ = 6.20, *p* = 0.016; (13) *F*_(1,11)_ = 7.48, *p* = 0.019; (14) *F*_(1,11)_ = 6.11, *p* = 0.031; (15) *F*_(1,11)_ = 8.27, *p* = 0.015; (16) *F*_(1,60)_ = 5.14, *p* = 0.027; (17) *F*_(1,11)_ = 93.50, *p* = 10^−5^; (18) *F*_(1,11)_ = 119.86, *p* < 10 − 6; (19) *F*_(1,11)_ = 42.94, *p* = 4.1 × 10^−5^; (20) *F*_(1,20)_ = 3.34, *p* = 0.082; (21) *F*_(1,21)_ = 4.73, *p* = 0.041; (22) *F*_(1,21)_ = 9.44, *p* = 0.0058; (23) *F*_(1,21)_ = 5.82, *p* = 0.025; (24) *F*_(1,23)_ = 3.39, *p* = 0.079; (25) *F*_(1,23)_ = 3.61, *p* = 0.07; (26) *F*_(1,22)_ = 5.062, *p* = 0.035; (27) *F*_(1,22)_ = 6.62, *p* = 0.017.

### Alterations in the Volume of Amygdala and PFC

**Figure 4.**
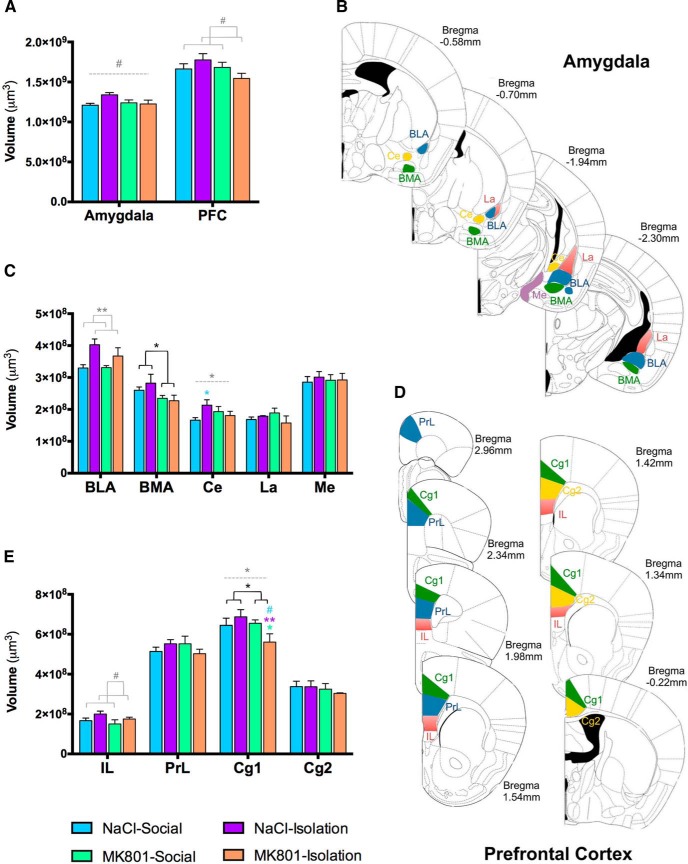
Volumetric analysis. Treatment, rearing, and the interaction of both treatments influenced the volume of the amygdala and the PFC. Although the total volume of both regions was not significantly affected (***A***), the volume of some nuclei of the amygdala (***B***, ***C***) and some regions of the PFC (***D***, ***E***) was affected by treatment (BMA, Cg1), rearing (BLA, IL), or their interaction (Ce, Cg1). Horizontal lines in graphs represent trends and statistically significant effects of MK801 treatment (black), rearing (gray), or interaction (gray, dashed) in a two-way ANOVA. # 0.1 > *p* > 0.05, *p < 0.05, **p < 0.01, ***p < 0.001. Colored symbols in bars represent trends and statistically significant differences among groups after *post hoc* analysis: # 0.1 > *p* > 0.05, *p < 0.05, **p < 0.01, ***p < 0.001. ***B***, ***D***, Schematic representations of the regions that were measured in our volumetric study (amygdala: all brain slices from bregma −0.58 to −2.30 mm; PFC: all brain slices from bregma +2.96 to −0.22 mm).

We have performed volumetric analyses to test whether the different interventions result in changes in the volumes of the amygdala and the PFC, as some studies have described in schizophrenic patients (Jaaro-Peled et al. 2010; Levitt et al. 2010; Fig. 4). Rearing in social isolation increased the volume of the BLA (Fig. 4*B*,*C*) and induced a trend toward an increase in the volume of the infralimbic (IL) cortex (Fig. 4*D*,*E*). By contrast, MK-801 treatment decreased the volume of the basomedial amygdala (BMA) (Fig. 4*B*,*C*) and the cingulate 1 (Cg1) cortex (Fig. 4*D*,*E*). A significant effect of the interaction of both interventions could also be observed in the central (Ce) nuclei of the amygdala (Fig. 4*B*,*C*) and the Cg1 cortex (Fig. 4*D*,*E*). When performing *post hoc* analyses, the isolated group of animals showed a significant increase in the volume of the Ce amygdala compared with the control group and double-hit mice showed a significant decrease in the volume of the Cg1 cortex compared with isolated or MK-801-treated animals (Fig. 4*C*,*E*; Table 2).

### Structural Alterations of E/I Neurons

**Figure 5.**
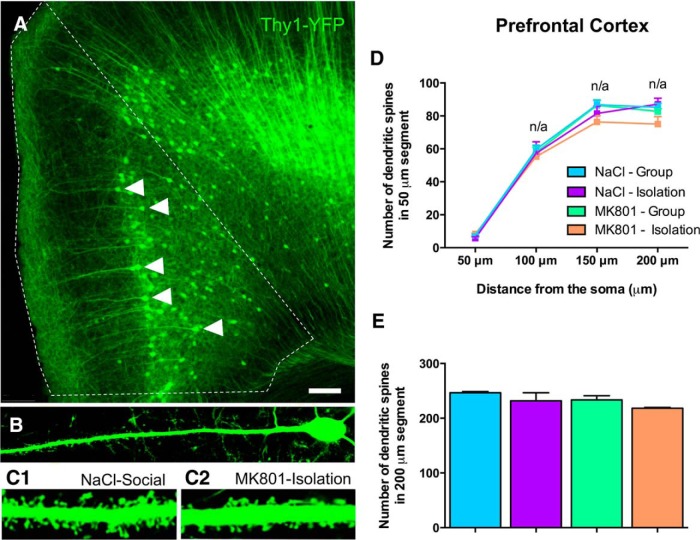
Structural analysis of pyramidal neurons. *A*, Panoramic view of the PFC of THY1-YFP expressing mice. Arrowheads point to fluorescent pyramidal neurons. ***B***, Representative confocal image of a pyramidal neuron from these animals. ***C1*** (NaCl-Group) and ***C2*** (MK801-Isolation) represent sections of the spiny apical dendrites of these pyramidal neurons. ***D***, ***E***, Graphs showing the results of the analysis of dendritic spine density. ***D***, The segmented analysis of the spine densities only showed a significant interaction effect in the first segment (0–50 µm of distance from the soma; Table 3) but no statistically significant differences among groups where observed after *post hoc* comparison. Despite the significant effects of treatment or rearing in some of the other segments (Table 3), the interaction (two-way ANOVA) was not significant in any of them and, therefore, *post hoc* comparisons were not applicable (n/a). ***E***, No statistical significant effects of treatment, rearing, or their interaction (two-way ANOVA) were observed when analyzing the spines density of the total length of the dendrite (200 µm). Scale bars: ***A***, 100 µm; ***B***, 20 µm; ***C1***, ***C2***, 2.5 µm.

Because these changes in the volume of the different regions studied could be due to changes in the structure of neurons, particularly of dendritic remodeling, we first analyzed spine density in PFC pyramidal neurons. Unfortunately, the characteristics of the strain (Porrero et al. 2010) do not allow for the analysis of dendritic arborization in these PFC neurons or any type of structural analysis in amygdaloid excitatory neurons. Treatment, rearing and their interaction affected the density of spines in different dendritic segments of pyramidal neurons in the PFC (Table 3). There was a decrease in the density of spines in the whole length of the dendrite, in double-hit mice compared with controls, but it was considered nonsignificant because of the lack of interaction between factors (Fig. 5*E*).

**Table 3.** Summary of results (part II)

Parameter	Main effects (two-way ANOVA)			Group differences (from NaCl-Social)		
	Treatment	Rearing	Interaction	NaCl-Isolation	MK801-Social	MK801-Isolation
**Structure of pyramidal neurons** (Fig. 5)						
Dendritic spines						
PFC						
50	—	—	✓^(28)^	—	—	—
100	—	—	—	n/a	n/a	n/a
150	—	✓^(29)^	—	n/a	n/a	n/a
200	✓^(30)^	—	—	n/a	n/a	n/a
Total	—	—	—	n/a	n/a	n/a
**Structure of interneurons** (Fig. 6)						
Dendritic arborization						
Amygdala						
20	—	#^(31)^	✓^(32)^	—	—	—
40	—	✓^(33)^	✓^(34)^	—	—	↑*
60	✓^(35)^	✓^(36)^	✓^(37)^	—	—	↑***
80	✓^(38)^	✓^(39)^	✓^(40)^	—	—	↑*
100	✓^(41)^	✓^(42)^	#^(43)^	n/a	n/a	n/a
120	✓^(44)^	✓^(45)^	✓^(46)^	—	—	↑*
140	#^(47)^	—	—	n/a	n/a	n/a
160	—	—	—	n/a	n/a	n/a
Total	✓^(48)^	✓^(49)^	✓^(50)^	—	—	↑***
PFC						
20	—	—	—	n/a	n/a	n/a
40	—	—	—	n/a	n/a	n/a
60	—	—	—	n/a	n/a	n/a
80	—	—	—	n/a	n/a	n/a
100	—	—	—	n/a	n/a	n/a
120	—	—	—	n/a	n/a	n/a
140	—	—	—	n/a	n/a	n/a
160	—	—	—	n/a	n/a	n/a
Total	—	—	—	n/a	n/a	n/a
Dendritic spines						
Amygdala						
50	—	—	—	n/a	n/a	n/a
100	—	—	—	n/a	n/a	n/a
150	—	#^(51)^	#^(52)^	n/a	n/a	n/a
Total	—	—	—	n/a	n/a	n/a
PFC						
50	#^(53)^	✓^(54)^	#^(55)^	n/a	n/a	n/a
100	✓^(56)^	✓^(57)^	✓^(58)^	↑*	↑**	↑**
150	✓^(59)^	✓^(60)^	—	n/a	n/a	n/a
Total	✓^(61)^	✓^(62)^	✓^(63)^	↑**	↑***	↑***

Symbols: (✓) statistically significant effect (two-way ANOVA); (—) no statistically significant effect (two-way ANOVA) or difference from NaCl-Social group (*post hoc*); (↑) increase; (n/a) not applicable; (↓) decrease; *p < 0.05; **p < 0.01; ***p < 0.001; #0.10 ≥ *p* ≥ 0.05.

*F* and *p* values: (28) *F*_(1,15)_ = 6.29, *p* = 0.024; (29) *F*_(1,15)_ = 4.88, *p* = 0.043; (30) *F*_(1,18)_ = 4.90, *p* = 0.043 (31) *F*_(1,19)_ = 3.81, *p* = 0.066; (32) *F*_(1,19)_ = 4.72, *p* = 0.043; (33) *F*_(1,19)_ = 9.02, *p* = 0.007; (34) *F*_(1,19)_ = 12.58, *p* = 0.002; (35) *F*_(1,18)_ = 7.53, *p* = 0.013; (36) *F*_(1,18)_ = 23.53, *p* = 12.8 × 10^−5^; (37) *F*_(1,18)_ = 12.62, *p* = 0.002; (38) *F*_(1,19)_ = 4.64, *p* = 0.044; (39) *F*_(1,19)_ = 6.55, *p* = 0.019; (40) *F*_(1,19)_ = 10.23, *p* = 0.005; (41) *F*_(1,20)_ = 4.43, *p* = 0.048; (42) *F*_(1,20)_ = 8.79, *p* = 0.008; (43) *F*_(1,20)_ = 3.27, *p* = 0.086; (44) *F*_(1,20)_ = 5.73, *p* = 0.027; (45) *F*_(1,20)_ = 5.32, *p* = 0.032; (46) *F*_(1,20)_ = 14.63, *p* = 0.0011; (47) *F*_(1,20)_ = 4.34, *p* = 0.050; (48) *F*_(1,20)_ = 8.31, *p* = 0.009; (49) *F*_(1,20)_ = 14.29, *p* = 0.001; (50) *F*_(1,20)_ = 18.23, *p* = 37.5 × 10^−5^; (51) *F*_(1,22)_ = 3.79, *p* = 0.064; (52) *F*_(1,22)_ = 3.34, *p* = 0.081; (53) *F*_(1,22)_ = 3.74, *p* = 0.066; (54) *F*_(1,22)_ = 4.53, *p* = 0.045; (55) *F*_(1,22)_ = 3.31, *p* = 0.083; (56) *F*_(1,22)_ = 11.72, *p* = 0.002; (57) *F*_(1,22)_ = 5.50, *p* = 0.029; (58) *F*_(1,22)_ = 5.18, *p* = 0.033; (59) *F*_(1,22)_ = 5.50, *p* = 0.028; (60) *F*_(1,22)_ = 7.43, *p* = 0.012; (61) *F*_(1,22)_ = 21.08, *p* = 14.2 × 10^−5^; (62) *F*_(1,22)_ = 6.27, *p* = 0.020; (63) *F*_(1,22)_ = 10.18, *p* = 0.004.

**Figure 6.**
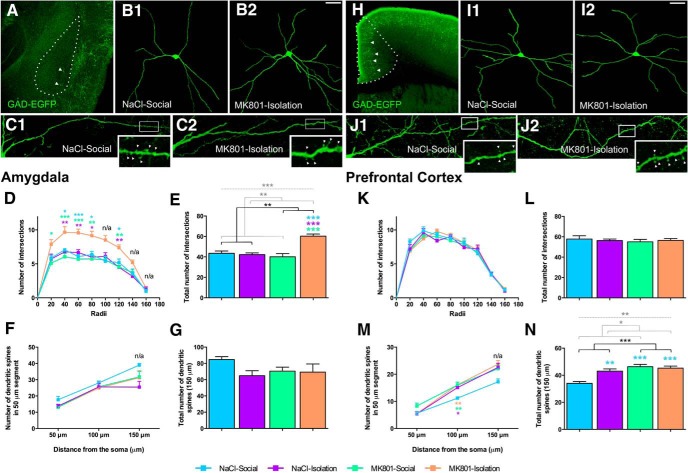
Structural analysis of interneurons. Panoramic view of the amygdala (***A***) and PFC (***H***) of GIN mice. ***B1***, ***B2***, ***I1***, ***I2***, Representative 3D reconstructions of the dendritic arbor of GAD-EGFP expressing interneurons. Double-hit mice showed increased dendritic arborization (number of intersections in the Sholl analysis) in interneurons form the amygdala (***D***, ***E***) but not from the PFC (***K***, ***L***). ***C1***, ***C2***, ***J1***, ***J2***, Representative images of spiny dendrites from GAD-EGFP expressing interneurons. Insets are magnified views of the squared sections of their respective images. Arrowheads point to dendritic spines. Analysis of the dendritic spines showed increased density in the PFC (***M***, ***N***) but not in the amygdala (***F***, ***G***) of double-hit mice. Horizontal lines in graphs (***E***, ***N***) represent statistically significant effects of MK801 treatment (black), rearing (gray), or interaction (gray, dashed) in a two-way ANOVA. *p < 0.05, **p < 0.01, ***p < 0.001. Colored symbols (***D***, ***E***, ***M***, ***N***) represent statistically significant differences among groups after *post hoc* analysis: *p < 0.05, **p < 0.01, ***p < 0.001. If the interaction (two-way ANOVA) was not significant, *post hoc* comparisons were not applicable (n/a). Scale bars: ***A***, ***H***, 800 µm; ***B***, ***I***, 40 µm; ***C***, ***J***, 14 µm; ***C***, ***J*** (insets), 2 µm.

Previous work from different laboratories has shown that the structure of interneurons is also susceptible to change in different paradigms, including chronic stress (Gilabert-Juan et al. 2011, 2013b). For this reason, we have also evaluated the structure of a subpopulation of these inhibitory neurons (Oliva et al. 2000). Treatment, rearing, and their interaction affected the total number of dendritic intersections in interneurons of the amygdala and the density of dendritic spines in interneurons of the PFC (Fig.6; Table 3). In particular, interneurons from double-hit mice showed significantly increased arborization in the amygdala (Fig.6*B1*,*B2*,*D*,*E*) and significantly increased density of dendritic spines in the PFC (Fig. 6*J1*,*J2*,*M*,*N*) compared with the control group.

### Decreased E/I Ratio in the Amygdala but Increased in the PFC

**Figure 7.**
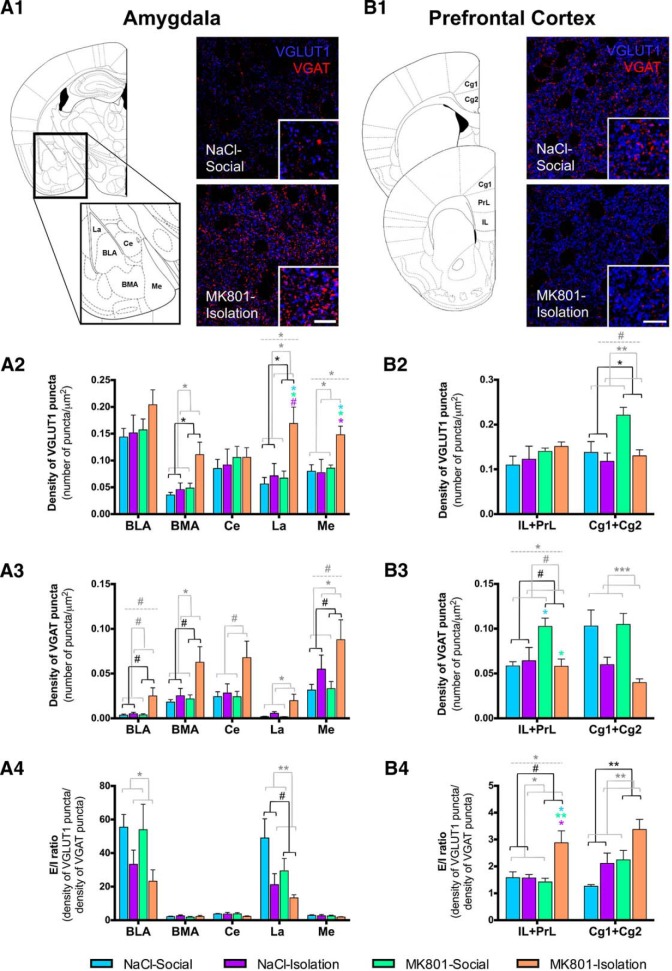
Excitatory and inhibitory neurotransmission. Schematic view of all the analyzed regions (***A1***, amygdala; ***B1***, PFC). Right, Representative confocal images of excitatory (blue, VGLUT1) and inhibitory (red, VGAT) puncta in the neuropil of NaCl-Social and MK801-Isolated animals. Scale bars: images, 16 µm; insets, 6 µm. Significant differences in VGLUT1 puncta density (***A2***, ***B2***), VGAT puncta density (***A3***, ***B3***), and E/I ratio (***A4***, ***B4***) among groups were observed. Horizontal lines in graphs represent trends and statistically significant effects of MK801 treatment (black), rearing (gray), or interaction (gray, dashed) in a two-way ANOVA. # 0.1 > *p* > 0.05, *p < 0.05, **p < 0.01, ***p < 0.001. Colored symbols in bars represent trends and statistically significant differences among groups after *post hoc* analysis: # 0.1 > *p* > 0.05, *p < 0.05, **p < 0.01, ***p < 0.001.

To have another readout of the putative alterations in inhibitory networks and the balance of excitatory versus inhibitory neurotransmission, we have studied with immunohistochemistry the expression of the GABA and glutamate vesicular transporters (VGAT and VGLUT1). Almost all nuclei/regions of the amygdala and PFC showed significant effects of treatment, rearing, or their interaction when analyzing the density of VGLUT1 and VGAT expressing puncta and E/I ratio (Table 4). Regarding excitatory puncta (VGLUT1), double-hit animals showed increased density in the La and Me nuclei of the amygdala (Fig. 7*A1*,*A2*) but no changes in any of the regions of the PFC (Fig. 7*B1*,*B2*). These animals also showed decreased density of VGAT puncta in the Cg1-Cg2 region of the PFC (Fig. 7*B1,B3* ). The E/I ratio was significantly increased in the PFC of double-hit animals (IL + PrL; (Fig. 7B4).

**Table 4.** Summary of results (part III)

Parameter	Main effects (two-way ANOVA)			Group differences (from NaCl-Social)		
	Treatment	Rearing	Interaction	NaCl-Isolation	MK801-Social	MK801-Isolation
**Density of neuropil puncta** (Fig. 7)						
VGLUT1 (Amygdala)						
Central	—	—	—	n/a	n/a	n/a
Lateral	✓^(64)^	✓^(65)^	✓^(66)^	—	—	↑*
Basolateral	—	—	—	n/a	n/a	n/a
Basomedial	✓^(67)^	✓^(68)^	#^(69)^	n/a	n/a	n/a
Medial	#^(70)^	✓^(71)^	✓^(72)^	—	—	↑*
VGLUT1 (PFC)						
IL+PrL	—	—	—	n/a	n/a	n/a
Cg1+Cg2	✓^(73)^	✓^(74)^	#^(75)^	n/a	n/a	n/a
VGAT (Amygdala)						
Central	—	#^(76)^	—	n/a	n/a	n/a
Lateral	—	✓^(77)^	—	n/a	n/a	n/a
Basolateral	#^(78)^	#^(79)^	#^(80)^	n/a	n/a	n/a
Basomedial	#^(81)^	✓^(82)^	—	n/a	n/a	n/a
Medial	#^(83)^	✓^(84)^	#^(85)^	n/a	n/a	n/a
VGAT (PFC)						
IL+PrL	#^(86)^	#^(87)^	✓^(88)^	—	↑*	—
Cg1+Cg2	—	✓^(89)^	—	n/a	n/a	n/a
**E/I Balance** (Fig. 7)						
Amygdala						
Central	—	—	—	n/a	n/a	n/a
Lateral	#^(90)^	✓^(91)^	—	n/a	n/a	n/a
Basolateral	—	✓^(92)^	—	n/a	n/a	n/a
Basomedial	—	—	—	n/a	n/a	n/a
Medial	—	—	—	n/a	n/a	n/a
PFC						
IL+PrL	#^(93)^	✓^(94)^	✓^(95)^	—	—	↑*
Cg1+Cg2	✓^(96)^	✓^(97)^	—	n/a	n/a	n/a

Symbols: (✓) statistically significant effect (two-way ANOVA); (-) no statistically significant effect (two-way ANOVA) or difference from NaCl-Social group (*post hoc*); (↑) increase; (n/a) not applicable; (↓) decrease; *p < 0.05; **p < 0.01; ***p < 0.001; #0.10 ≥ *p* ≥ 0.05.

*F* and *p* values: (64) *F*_(1,29)_ = 4.58, *p* = 0.041; (65) *F*_(1,29)_ = 7.12, *p* = 0.012; (66) *F*_(1,29)_ = 4.20, *p* = 0.049; (67) *F*_(1,28)_ = 4.89, *p* = 0.036; (68) *F*_(1,28)_ = 6.08, *p* = 0.020; (69) *F*_(1,28)_ = 3.17, *p* = 0.086; (70) *F*_(1,29)_ = 3.74, *p* = 0.063; (71) *F*_(1,29)_ = 4.32, *p* = 0.047; (72) *F*_(1,29)_ = 5.01, *p* = 0.033; (73) *F*_(1,19)_ = 6.31, *p* = 0.021; (74) *F*_(1,19)_ = 8.44, *p* = 0.009; (75) *F*_(1,19)_ = 3.54, *p* = 0.075; (76) *F*_(1,29)_ = 3.45, *p* = 0.074; (77) *F*_(1,28)_ = 5.28, *p* = 0.029; (78) *F*_(1,29)_ = 3.09, *p* = 0.089; (79) *F*_(1,29)_ = 3.88, *p* = 0.058; (80) *F*_(1,29)_ = 2.91, *p* = 0.097; (81) *F*_(1,29)_ = 3.16, *p* = 0.086; (82) *F*_(1,29)_ = 4.29, *p* = 0.047; (83) *F*_(1,29)_ = 3.47, *p* = 0.073; (84) *F*_(1,29)_ = 5.88, *p* = 0.022; (85) *F*_(1,29)_ = 3.05, *p* = 0.091; (86) *F*_(1,18)_ = 3.75, *p* = 0.069; (87) *F*_(1,18)_ = 3.88, *p* = 0.065; (88) *F*_(1,18)_ = 6.53, *p* = 0.020; (89) *F*_(1,19)_ = 21.12, *p* = 2 × 10^−4^; (90) *F*_(1,29)_ = 3.55, *p* = 0.070; (91) *F*_(1,29)_ = 9.01, *p* = 0.005; (92) *F*_(1,28)_ = 6.75, *p* = 0.015; (93) *F*_(1,18)_ = 3.93, *p* = 0.063; (94) *F*_(1,18)_ = 6.18, *p* = 0.023; (95) *F*_(1,18)_ = 6.39, *p* = 0.021; (96) *F*_(1,18)_ = 10.72, *p* = 0.004; (97) *F*_(1,18)_ = 8.33, *p* = 0.0098.

### Expression of GAD67, SYN, and PSA-NCAM

**Figure 8.**
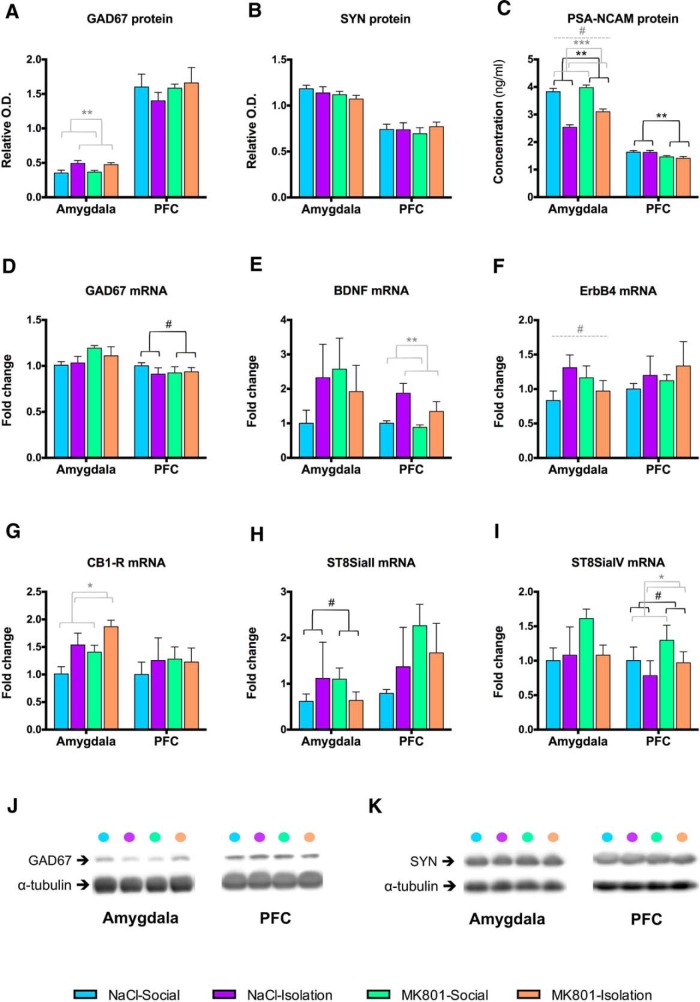
Protein and gene expression studies. Bar graphs showing the presence or lack of effect of the treatment, rearing, or their interaction in GAD67, SYN, and PSA-NCAM protein expression (***A***–***C***) and in GAD67, BDNF, ErbB4, CB1-R, ST8SiaII, and ST8SiaIV mRNA levels (***D***–***I***). Horizontal lines in graphs represent trends and statistically significant effects of MK801 treatment (black), rearing (gray) or interaction (gray, dashed) in a two-way ANOVA. # 0.1 > *p* > 0.05, *p < 0.05, **p < 0.01, ***p < 0.001. ***J*** & ***K***: Representative bands from immunoblots for GAD67 (***J***) and SYN (***K***) in the amygdala and the PFC.

The differences that we have found in the structure of interneurons and in the E/I ratio prompted us to study the expression of molecules related to inhibitory neurotransmission and to the plasticity of interneuronal networks, such as the polysialylated form of the neural cell adhesion molecule (PSA-NCAM; Nacher et al. 2013). GAD67 protein expression in the amygdala was significantly influenced by rearing (increased as a consequence of social isolation) but double-hit animals failed to show significant differences compared with the control group (Table 5; Fig. 8*A*). Synaptophysin (SYN) protein expression in both regions and GAD67 expression in the PFC were not affected by any experimental condition (Table 5; Fig. 8*A*,*B*). PSA-NCAM expression was influenced by rearing (decreased expression in the amygdala after social isolation) and treatment (after MK-801, increased expression in the amygdala and decreased expression in the PFC) but not by their interaction (Table 5; Fig. 8*B*,*D*).

**Table 5.** Summary of results (part IV)

Parameter	Main effects (two-way ANOVA)			Group differences (from NaCl-Social)		
	Treatment	Rearing	Interaction	NaCl-Isolation	MK801-Social	MK801-Isolation
**Protein expression** (Fig.7)						
GAD67						
Amygdala	—	✓^(98)^	—	n/a	n/a	n/a
PFC	—	—	—	n/a	n/a	n/a
SYN						
Amygdala	—	—	—	n/a	n/a	n/a
PFC	—	—	—	n/a	n/a	n/a
PSA-NCAM						
Amygdala	✓^(99)^	✓^(100)^	#^(101)^	n/a	n/a	n/a
PFC	✓^(102)^	—	—	n/a	n/a	n/a
**Gene expression** (Fig.7)						
GAD67						
Amygdala	—	—	—	n/a	n/a	n/a
PFC	#^(103)^	—	—	n/a	n/a	n/a
BDNF						
Amygdala	—	—	—	n/a	n/a	n/a
PFC	—	✓^(104)^	—	n/a	n/a	n/a
ErbB4						
Amygdala	—	—	#^(105)^	n/a	n/a	n/a
PFC	—	—	—	n/a	n/a	n/a
CB1-R						
Amygdala	—	✓^(106)^	—	n/a	n/a	n/a
PFC	—	—	—	n/a	n/a	n/a
ST8SiaII						
Amygdala	#^(107)^	—	—	n/a	n/a	n/a
PFC	—	—	—	n/a	n/a	n/a
ST8SiaIV						
Amygdala	—	—	—	n/a	n/a	n/a
PFC	#^(108)^	✓^(109)^	—	n/a	n/a	n/a

Symbols: (✓) statistically significant effect (two-way ANOVA); (—) no statistically significant effect (two-way ANOVA) or difference from NaCl-Social group (*post hoc*); (↑) increase; (n/a) not applicable; (↓) decrease; (*) *p* < 0.05; (**) *p* < 0.01; (***) *p* < 0.001; (#) 0.10 ≥ *p* ≥ 0.05.

*F* and *p* values: (98) *F*_(1,19)_ = 11.74, *p* = 0.003; (99) *F*_(1,25)_ = 11.58, *p* = 0.002; (100) *F*_(1,25)_ = 107.23, *p* < 10^−10^; (101) *F*_(1,25)_ = 4.02, *p* = 0.056; (102) *F*_(1,32)_ = 9.863, *p* = 0.004; (103) *F*_(1,25)_ = 3.94, *p* = 0.058; (104) *F*_(1,26)_ = 13.51, *p* = 0.001; (105) *F*_(1,25)_ = 4.10, *p* = 0.054; (106) *F*_(1,26)_ = 5.52, *p* = 0.027; (107) *F*_(1,20)_ = 3.07, *p* = 0.095; (108) *F*_(1,25)_ = 4.12, *p* = 0.053; (109) *F*_(1,25)_ = 5.06, *p* = 0.033.

### Rearing Influences *BDNF, CB1-R*, and *St8SiaIV* Gene Expression

We have also studied the expression of different genes involved in the development and regulation of inhibitory networks, including the enzymes responsible for PSA synthesis. Among all the studied genes, a significant effect of rearing was found when studying BDNF (increased after social isolation) and ST8SiaIV mRNA expression (decreased after social isolation) in the PFC and CB1-R mRNA expression (increased after social isolation) in the amygdala (Table 5; Fig. 8*D*–*I*).

### Number of PNNs and PV-Expressing Neurons Surrounded by PNNs

**Figure 9.**
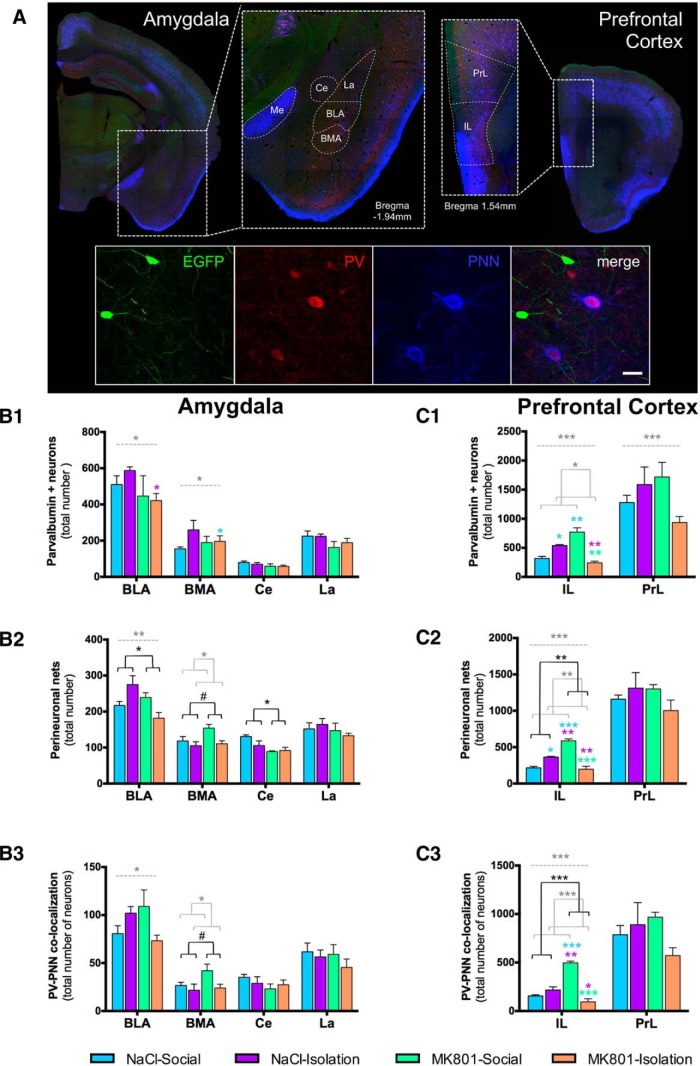
Expression of PNNs in PV-immunoreactive neurons. **A**, Representative confocal images showing the distribution of PV (red) and PNNs (blue) in the different nuclei of the amygdala (left) and regions of the PFC (right) of GIN mice (GAD-GFP, green). The squared section in the panoramic images was 2× magnified to show the different nuclei and areas that were analyzed. In a detailed view (below), two PV-immunoreactive neurons expressing PNN can be observed. GAD-GFP expressing interneurons do not express PNN. Scale bars: panoramic views, 875 µm; detailed view, 25 µm. ***B***, ***C***, Treatment, rearing, or their interaction affected the total number of PV neurons (***B1***, ***C1***), PNNs (***B2***, ***C2***), or their colocalization (***B3***, ***C3***) in some nuclei of the amygdala and regions of the PFC. Horizontal lines in graphs represent trends and statistically significant effects of MK801 treatment (black), rearing (gray), or interaction (gray, dashed) in a two-way ANOVA. # 0.1 > *p* > 0.05, *p < 0.05, **p < 0.01, ***p < 0.001. Colored symbols in bars represent trends and statistically significant differences among groups after *post hoc* analysis: # 0.1 > *p* > 0.05, *p < 0.05, **p < 0.01, ***p < 0.001.

Different components of the extracellular matrix can play a role in the E/I imbalance and the remodeling of inhibitory networks observed in our study. Of particular interest are PNNs, which are abundant around PV-expressing interneurons (Härtig et al. 1992). The composition of these PNN is regulated dynamically in response to aversive experiences, such as fear or stress, and alterations in the expression of PNNs have been observed in the PFC of schizophrenic patients (Berretta et al. 2015). In our study, effects of treatment, isolation, and their interaction were found in different regions of the mPFC and the amygdala. Specifically, in the infralimbic cortex, both isolated, and especially MK-801-treated animals, showed a significant increase in the number of PV-expressing neurons, PNNs, and PV-expressing neurons surrounded by PNNs (only MK-801; Table 6; Fig. 9). By contrast, in this region there was a tendency for a decrease in these parameters in the double-hit mice.

**Table 6.** Summary of results (part V)

Parameter	Main effects (two-way ANOVA)			Group differences (from NaCl-Social)		
	Treatment	Rearing	Interaction	NaCl-Isolation	MK801-Social	MK801-Isolation
**PV+ neurons** (Fig. 9)						
Amygdala						
Total	—	—	#^(110)^	n/a	n/a	n/a
Central	—	—	—	n/a	n/a	n/a
Lateral	—	—	—	n/a	n/a	n/a
Basolateral	—	—	✓^(111)^	—	—	—
Basomedial	—	—	✓^(112)^	—	—	↑*
PFC						
Total	—	—	✓^(113)^	—	—	—
IL	—	✓^(114)^	✓^(115)^	↑*	↑**	—
PrL	—	—	✓^(116)^	—	—	—
**PNNs** (Fig. 9)						
Amygdala						
Total	✓^(117)^	—	✓^(118)^	—	—	—
Central	✓^(119)^	—	—	n/a	n/a	n/a
Lateral	—	—	—	n/a	n/a	n/a
Basolateral	✓^(120)^	—	✓^(121)^	—	—	—
Basomedial	#^(122)^	✓^(123)^	—	n/a	n/a	n/a
PFC						
Total	—	—	✓^(124)^	—	↑^**^	—
IL	✓^(125)^	✓^(126)^	✓^(127)^	↑*	↑***	—
PrL	—	—	—	n/a	n/a	n/a
**PV-PNN colocalization** (Fig. 9)						
Amygdala						
Total	—	✓^(128)^	✓^(129)^	—	↑#	—
Central	—	—	—	n/a	n/a	n/a
Lateral	—	—	—	n/a	n/a	n/a
Basolateral	—	—	✓^(130)^	—	—	—
Basomedial	#^(131)^	✓^(132)^	—	n/a	n/a	n/a
PFC						
Total	—	#^(133)^	✓^(134)^	—	↑**	↓#
IL	✓^(135)^	✓^(136)^	✓^(137)^	—	↑***	—
PrL	—	—	—	n/a	n/a	n/a

Symbols: (✓) statistically significant effect (two-way ANOVA); (—) no statistically significant effect (two-way ANOVA) or difference from NaCl-Social group (*post hoc*); (↑) increase; (n/a) not applicable; (↓) decrease; *p < 0.05; **p < 0.01; ***p < 0.001; #0.10 ≥ *p* ≥ 0.05.

*F* values and *p* values: (110) *F*_(1,21)_ = 3.99, *p* = 0.059; (111) *F*_(1,18)_ = 7.06, *p* = 0.016; (112) *F*_(1,20)_ = 4.80, *p* = 0.041; (113) *F*_(1,14)_ = 12.82, *p* = 0.003; (114) *F*_(1,15)_ = 7.22, *p* = 0.017; (115) *F*_(1,15)_ = 52.97, *p* = 3 × 10^−6^; (116) *F*_(1,15)_ = 7.03, *p* = 0.018; (117) *F*_(1,20)_ = 5.648, *p* = 0.028; (118) *F*_(1,20)_ = 6.70, *p* = 0.016; (119) *F*_(1,18)_ = 10.19, *p* = 0.005; (120) *F*_(1,21)_ = 4.91, *p* = 0.038; (121) *F*_(1,21)_ = 12.96, *p* = 0.002; (122) *F*_(1,20)_ = 3.39, *p* = 0.08; (123) *F*_(1,20)_ = 6.4, *p* = 0.02; (124) *F*_(1,14)_ = 10.55, *p* = 0.006; (125) *F*_(1,15)_ = 12.93, *p* = 0.003; (126) *F*_(1,15)_ = 17.92, *p* = 0.001; (127) *F*_(1,15)_ = 88.68, *p* < 10 − 7; (128) *F*_(1,19)_ = 10.95, *p* = 0.004; (129) *F*_(1,19)_ = 13.36, *p* = 0.002; (130) *F*_(1,19)_ = 6.53, *p* = 0.019; (131) *F*_(1,21)_ = 2.99, *p* = 0.098; (132) *F*_(1,21)_ = 4.97, *p* = 0.037; (133) *F*_(1,13)_ = 3.61, *p* = 0.08; (134) *F*_(1,13)_ = 15.49, *p* = 0.002; (135) *F*_(1,15)_ = 19.87, *p* = 4.61 × 10^−4^; (136) *F*_(1,15)_ = 47.52, *p* = 5 × 10^−6^; (137) *F*_(1,15)_ = 87.65, *p* < 10^−7^.

## Discussion

In the present study, we report alterations in behavior, neuronal structural plasticity, and E/I neurotransmission in mice subjected to chronic early-life stress, consisting in a social isolation rearing from the age of weaning until adulthood. A group of animals (double-hit) was also subjected to a perinatal injection of the NMDAR antagonist MK801 to interfere with the last stages of neocortical development: the activation of the NMDA receptor is essential for neuronal differentiation, migration, and survival (Jansson and Åkerman, 2014) and perinatal treatments with NMDAR antagonists trigger apoptosis, specially in the frontal neocortex (Ikonomidou et al. 1999; Wang and Johnson, 2005).

Both interventions, isolation and MK801 treatment, have been considered animal models for schizophrenia. We have found that, especially, the isolation rearing induces a wide spectrum of alterations, including changes in neurochemical, behavioral, and structural parameters. These changes affect particularly interneurons and some of them are significant when this aversive experience is combined with the neurodevelopmental intervention. These effects are the consequence of prolonged isolation stress during a period in which the circuits are already stablished, although they still display a high degree of plasticity. Alterations on the structure and neurotransmission of interneurons can be induced even in young-adult animals when they are subjected to chronic stress (Gilabert-Juan et al. 2011). By contrast, the single injection of MK801 in P7 is a procedure that disturbs acutely ongoing neurodevelopmental processes, particularly in the frontal cortex, such as neuronal migration, neurite extension, or synaptogenesis. This is an important time point for the maturation of inhibitory circuits. When animals are subjected to both alterations (MK801 injection and social isolation), we observe a cumulative effect, especially on the structure of interneurons. Probably the early effect of MK801 disturbs the construction of interneuronal morphology and connectivity. In animals reared in group, these alterations are probably compensated during infancy and puberty: for instance, in the proportion of PV-expressing neurons surrounded by PNNs in the infralimbic cortex, the significant effects of the MK801 injection appear to be reverted by the social isolation. However, if the animals are subjected to the prolonged isolation stress, the impact of this aversive experience may prevent this compensation. The social isolation stress by itself seems to be enough to produce significant changes in many parameters, but in others, like some anxiety-like and locomotor behaviors, this aversive experience is not enough to render significant alterations. Apparently, these changes only appear when the stressful event operates on a previously altered substrate, such as the subtly modified circuitry of MK801-injected animals.

### Behavioral Alterations

Anxiety, hyperactivity, and working memory deficits are frequently reported in patients suffering from schizophrenia (Pallanti et al. 2013; Pallanti and Salerno, 2015; Van Snellenberg et al. 2016) and in several animal models of this disease (Jones et al. 2011; Lett et al. 2014). In our study, we used the hole-board apparatus to analyze these behaviors (Karl et al. 2008; Castilla-Ortega et al. 2010; Torres-García et al. 2012). Our results on GIN mice show that rearing in isolation induced anxiety-like behavior and locomotor hyperactivity. Therefore, the functional consequences of the molecular and structural changes affecting only the double-hit GIN mice are not yet clear. The analysis of Thy1 mice also showed these effects of rearing, but in this strain also revealed significant differences in double-hit animals compared with controls. These behavioral abnormalities may be a long-term consequence of the impairments in brain development that are induced by post-weaning social deprivation, as other authors reported before (Koike et al. 2009; Jones et al. 2011; Hickey et al. 2012; Jiang et al. 2013). No changes in exploratory behavior were observed in any of the strains when measured in the hole-board; this test can be considered as an initial basic screen for working memory (Karl et al. 2008).

Studies on schizophrenia models based on NMDAR hypofunction during early postnatal development have yielded contradictory results; whereas some authors described hyperlocomotion, anxiety-related behaviors, and working memory abnormalities after these treatments (Andersen and Pouzet, 2004; Bubeníková-Valesová et al. 2008; Belforte et al. 2010; Nozari et al. 2015), others failed to find them (Bubeníková-Valesová et al. 2008; Rompala et al. 2013).

### Changes in Brain Volume and Neuronal Structure

Numerous studies in humans and animal models of schizophrenia have documented the presence of structural alterations in the BLA and PFC, including volume impairments (Jaaro-Peled et al. 2010; Levitt et al. 2010). Early-life stress also has dramatic effects on brain structure, especially on the amygdala (hypertrophy, increased activity, and dendritic arborization of excitatory neurons; for review, see Coplan et al. 2014). In our study, early-life stress, but not specifically the double-hit model, has a negative effect in the volume of the PFC (as described previously in rats, Gilabert-Juan et al. 2013a), but increases the volume of CeA. To our knowledge, there are no previous studies on the effects of social isolation rearing on these nuclei, although reductions in the volume of the MeA have been described (Cooke et al. 2000).

As these volumetric alterations may probably reflect structural changes in PFC and BLA neurons, we decided to analyze their structure. In fact, a previous Golgi study in rats found that social isolation rearing reduces the length of dendritic segments of pyramidal neurons in the BLA while increasing their complexity (Wang et al. 2012). This study also found reduced dendritic arborization and spine density in mPFC pyramidal neurons, similar to what has been found after chronic stress in adult rats (Radley et al. 2004, 2006). By contrast, also with data from schizophrenic patients and rat models (Jones et al. 2011; Flores et al. 2016), we have not observed a significant reduction in spine density of the PFC pyramidal neurons in isolated or double-hit mice. This controversy could be due to species differences. However, because there is a clear trend toward a decrease in the double-hit animals, it is possible that studies with higher number of animals would render significant results on this parameter.

An increase in dendritic spine density was found in PFC interneurons of isolated and double-hit mice, which may lead to an increased number of synaptic contacts on these interneurons. Future experiments should explore whether these new spines are active and receive excitatory and/or inhibitory contacts. We have also detected a significant increase in dendritic arborization in amygdaloid interneurons of the double-hit mice, also suggesting an increased surface for the reception of new synaptic contacts. Interestingly, the subpopulation of interneurons studied in the present paper, which mainly express somatostatin (Oliva et al. 2000), also exhibits hypertrophic changes when young adult mice are subjected to chronic stress (Gilabert-Juan et al. 2011, 2013b). These findings are especially relevant because, despite the abundant number of studies that point to interneurons as key players in neurodevelopmental mental disorders, no studies looking for dendritic abnormalities in interneurons, neither of the PFC nor the amygdala, have been conducted before.

### Alterations in Structural Plasticity and E/I Neurotransmission

The impairments in the morphology and connectivity of neurons may be mediated by changes in the expression of the plasticity-related molecule PSA-NCAM (Bonfanti, 2006; Rutishauser, 2008; Nacher et al. 2013). Post-weaning social isolation rearing in rats leads to increases in the expression of PSA-NCAM during adulthood, specially in the basolateral amygdaloid nucleus (Gilabert-Juan et al. 2012). Adult rats exposed to different physical stressors during adolescence show a similar increase in this nucleus (Tsoory et al. 2008). However, in the present experiment, both the isolated and the double-hit mice showed decreased expression of PSA-NCAM in the amygdala in adult life. These apparently contradictory results could be due to species differences and/or to a different impact of stress in different amygdaloid regions, because the expression of PSA-NCAM in the present study has been studied in the whole amygdala. In fact, similar reductions in the expression of this molecule have been found after chronic stress in the central amygdala of adult mice (Gilabert-Juan et al. 2011) and rats (Cordero et al. 2005). These findings are specially relevant for inhibitory circuits, because in the amygdala and PFC, PSA-NCAM is exclusively expressed by interneurons, and therefore is able to regulate directly its structure and connectivity (Nacher et al. 2013).

PNNs were reduced in the lateral nucleus of the amygdala (Pantazopoulos et al. 2010) and the PFC in schizophrenic patients (Mauney et al. 2013). Reduced labeling of PV neurons was also observed in these patients (Enwright et al. 2016). Moreover, a recent report has described a disruption of PNNs in the mPFC after in an animal model of this disorder (maternal immune activation; Paylor et al. 2016). Our study also reveals a similar, although nonsignificant, decrease in these parameters in the infralimbic cortex of the double-hit mice. This is in accordance with previous results on PV-expressing neurons in this prefrontocortical region using the same double-hit model in rats (Gilabert-Juan et al. 2013a). However, it has to be noted that opposite effects were observed in the MK-801 group of animals. We do not have an explanation yet for these effects and there are no previous studies looking at the effects of NMDAR antagonists on PNNs.

The precise balance between excitatory and inhibitory neurotransmission is crucial for the proper maturation of the neural circuitry during development. In fact, E/I imbalances in key brain regions have been proposed as underlying causes of some neurodevelopmental psychiatric disorders, including schizophrenia (Curley and Lewis, 2012; Lewis et al. 2012; Inan et al. 2013; Lin et al. 2013; Sun et al. 2013; Morishita et al. 2015). Our data support this hypothesis, demonstrating that early-life stress strongly influences E/I balance and that alterations in this balance are particularly evident in the PFC and the amygdala of double-hit mice. E/I balance can also be affected by changes in the expression of other molecules that influence the maturation of inhibitory circuits and that have also been related to schizophrenia. This is the case of BDNF (Sánchez-Huertas and Rico, 2011), ST8SiaIV (Nacher et al. 2010), and CB1-R (Volk and Lewis, 2010; den Boon et al. 2015). BDNF and ST8SiaIV mRNA levels in the PFC are increased and decreased, respectively, by early-life stress, whereas CB1-R mRNA levels in the amygdala are increased.

#### Concluding remarks and future perspectives

The present results suggest an important impact of stressful events in early life on inhibitory networks, both at the structural and the neurochemical level. We still do not know whether the changes in the structure (and probably in the connectivity) of interneurons precede or are the response to alterations in the excitatory neurotransmission. Future experiments evaluating different time points should clarify this matter. The present model may constitute a powerful resource for the study of the influence of early-life aversive experiences on the adult brain and to deepen further in the understanding of the neurobiological basis of mental disorders in which these experiences seem to play a critical role, such as schizophrenia. The model may constitute an interesting tool for testing new antipsychotic drugs or to understand how other genetic or environmental factors, particularly during the early-life stages, can impact on the development of this symptomatology. Future studies will have to expand the battery of behavioral tests, particularly those measuring prefrontal- and amygdaloid-related behaviors. Given the alterations found in the structure of interneurons and in some molecules related to inhibitory neurotransmission, it would also be interesting to analyze them in more detail.

## Synthesis

Reviewing Editor: Liisa Galea, University of British Columbia Decisions are customarily a result of the Reviewing Editor and the peer reviewers coming together and discussing their recommendations until a consensus is reached. When revisions are invited, a fact-based synthesis statement explaining their decision and outlining what is needed to prepare a revision will be listed below.

The authors have made a concerted effort to revise the manuscript in response to reviewer comments. The manuscript is much improved, especially concerning the statistical analysis. The improved language concerning the limited effect of the “double hit” is appreciated as well. The authors have added more data and enhanced the presentation of existing data. The figures, especially where immunohistochemistry is concerned, are very well done. However, there are still some concerns regarding the statistics, more explanation needed in the methods section, and interpretations within the discussion that remain to be addressed as listed below.

Methods:

There is no description of what “immobility” time is or “immobility episodes” (line 181-182). Given that distance travelled is likely a mirror image of immobility, only one of these variables should be used.

Working memory - this is not a well validated approach to studying working memory - which typically involves examining errors during a trial an animal may make. In this “task” there are no errors to make, the animal is simply exploring the novel environment. What is meant by “novel hole.” This is a measure of exploration but not necessarily working memory as is normally defined. It is suggested that this aspect is better described and not referred to as working memory but rather exploration (see lines 175, 183 +).

They have also not described what immobility is - and my guess is that it is just the opposite to moving so no wonder that both activity and immobility are significant.

Statistics:

In a number of places within the text the results are refered to as “altered” without giving a direction. For example: line 405 - “clearly influenced” - be specific what was clearly influenced and it what direction, line 414: “significant differences were found in most of the parameters for locomotion” please provide details..what differences, what direction and which parameters?; line 424 - “was affected.” Please be specific ; line 474: please describe direction of effect on GAD67; line 478 - PSA=NCAM was influenced by treatment in amygdala and PFC and rearing in the amygdala but the authors do not say in which direction.

Line 236. Line 393 - it is not known what is meant by statistics were performed by using the number of animals and the 'n.' What is the 'n'?

Why is genotype not in the analysis as another independent variable?

Line 393-396; lines 437-439 - it is possible to run pair-wise comparisons on comparisons of interest even if there is no significant interaction as long as you have an a priori reason to do so and you account for multiple comparisons (with a bonferroni for example).

Discussion:

The amount of space that is allotted to discussing findings that are not significantly altered by the “double hit” dilutes some of the interesting discussion that could be taking place. Given that there were limited effects of the double hit, this is somewhat understandable. Alternatively, one interesting issue that could be addressed is this: the MK-801 treatment and social isolation were administered in very different ways (acutely versus chronically) and at different times of brain development (early postnatal versus pre-pubertal onward). What might the authors be able to infer about brain development from the instances where there was no effect of the “double hit” beyond single treatment? What do the “double hit” findings tell us about brain development, especially concerning the findings of the interneuron structure? Additionally interesting is the finding that the “double hit” seems to reverse the effect of either treatment alone on the PVN-PNN colabeling. A discussion at this level would improve the manuscript and may enhance what can seem like a disjointed discussion at times.

Minor

Line 54 - 'since' should only be used when referring to time past. Replace with 'because'

Line 142 - 'born' should be 'birth'

line 424: “Double-hit' animals show a significant increase in the volume of the central amygdala when compared to 426 the control group (Fig. 4, table 2).” Could there be a typo in this sentence? If not, there is a disconnect in Figure 4, where there looks to be an increase in the Isolation alone group, including indication of significant difference by an asterisk.

Figure 8. Please provide figures of representative bands from immunoblots that were obtained for protein measurement.
